# Monitoring training load in youth soccer players: effects of a six-week preparatory training program and the association between external and internal loads

**DOI:** 10.5114/biolsport.2023.112094

**Published:** 2022-01-03

**Authors:** Sandra Lechner, Achraf Ammar, Omar Boukhris, Khaled Trabelsi, Jordan M Glenn, Jesper Schwarz, Omar Hammouda, Piotr Zmijewski, Hamdi Chtourou, Tarak Driss, Anita Hoekelmann

**Affiliations:** 1 Institute of Sport Science, Otto-von-Guericke University, 39106, Magdeburg, Germany; 2 Department of Training and Movement Science, Institute of Sport Science, Johannes Gutenberg-University Mainz, 55099 Mainz, Germany; 3 Interdisciplinary Laboratory in Neurosciences, Physiology and Psychology: Physical Activity, Health and Learning (LINP2), UPL, UFR STAPS (Faculty of Sport Sciences), Paris Nanterre University, Nanterre, France; 4 High Institute of Sport and Physical Education of Sfax, University of Sfax, Sfax, Tunisia; 5 Research Unit, “Physical Activity, Sport and Health”, UR18JS01, National Observatory of Sport, Tunis, Tunisia; 6 Research Laboratory: Education, Motricity, Sport and Health, EM2S, LR19JS01, University of Sfax, Sfax, Tunisia; 7 Department of Health, Exercise Science Research Center Human Performance and Recreation, University of Arkansas, Fayetteville, Arkansas, USA; 8 Braunschweiger Turn- und Sportverein Eintracht von 1895 e.V., Braunschweig, Lower Saxony, Germany; 9 “Research Laboratory, Molecular Bases of Human Pathology, LR19ES13, Faculty of Medicine, University of Sfax, Sfax, Tunisia; 10 Jozef Pilsudski University of Physical Education in Warsaw, Warsaw, Poland

**Keywords:** Youth football, External parameters, Internal parameters, Training monitoring

## Abstract

This study examined the effects of a six-week preparatory training program on physical performance and physiological adaptations in junior soccer players. Additionally, we investigated whether a relationship existed between external and internal loads. Youth soccer players (aged 16 years old) from a youth football academy participated in six weeks of pre-conditioning training. Wireless Polar Team Pro and Polar heart rate sensors (H10) were used to monitor physical performance indicators (sprint and acceleration scores, covered distance, maximum and average speed and duration), physiological responses (maximum and average heart rate [HR] and R-R interval, time in HR zones 4+5, and heart rate variability [HRV]), and training load score. Additionally, muscle status and rating of perceived exertion (RPE) scores were measured using digital questionnaires. Significant increases were observed in the majority of physical performance indicators [i.e., sprints (p = 0.015, ES = 1.02), acceleration (p = 0.014, ES = 1), total distance (p = 0.02, ES = 0.87), as well as maximum speed (p = 0.02, ES = 0.87)]. A trend towards improvement was observed in the remaining performance indicators (i.e., distance/min and avg speed; ES = 0.6), training load (ES = 0.2), muscle status (ES = 0.3)), and all physiological responses parameters (ES = 0.1 to 0.6). Significant correlations were found between the majority of external load parameters (i.e., performance indicators) and objective (i.e., physiological responses) and subjective (i.e., RPE, muscle status) internal load parameters (p < 0.001). The highest number of moderate-large correlations were registered between performance indicators and time in HR zone 4+5 (0.58 < r < 0.82), training load (0.53 < r < 0.83), average HR (0.50 < r < 0.87), maximal HR (0.51 < r < 0.54) and average R-R interval (0.58 < r < 0.76). HR zone 4+5, average and maximal HR, average R-R interval, and training load score may help control training parameters and reduce the risk of under- or over-training in youth soccer players. However, these conclusions should be confirmed and replicated in future studies with more diverse subject populations.

## INTRODUCTION

Soccer is one of the most popular sports in the world, and top-level performance depends on physical fitness, psychological factors, technical skills, and team tactics [1]. In order to best prepare players to perform well during a match, while reducing risk of injury, training load is often recorded and monitored [2]. It is important to measure the physical loads applied to each player (e.g., distance covered, accelerations), and the subsequent physiological response, such as heart rate (HR) or subjective level of effort. This difference between these parameters is referred to as external and internal load [3–5]; there are numerous parameters for both external and internal load that are used in training management.

Recording the loads in soccer is often completed via the monitoring of training. “Training monitoring” is the systematic collection of data describing the amount, intensity, or content of the training [6], and is considered an effective method for training control [7]. Furthermore, training control is the visualization and objectification of a training process using appropriate performance parameters and technical instruments [6]. Training control allows the trainer to compare planning and performance, with specific regard to the athlete’s age, gender and performance level [6]. Based on training monitoring’s data, it is decided daily whether an athlete is ready for training or competition in order to create an optimal balance between load and recovery, which is essential for improving athletic performance [8].

In addition, periodization is used to optimize load management [9, 10]. Weekly periodization occurs when players experience varied training loads on different training days; over a longer period the load is steadily increased over three weeks, reduced in the fourth week, and then subsequently increased again [11]. The reduction during the 4^th^ week, is referred to as tapering and increases game readiness [12, 13]. During the preparation phase, a gradual increase of the training load has been shown to reduce the risk of injury, while maintaining or even increasing performance throughout the season [11].

It is already established that there is a relationship between external and internal stress parameters [14, 15] as well as between objective and subjective internal exertion parameters [4]. In particular, significant correlations exist between athletes’ rating of perceived exertion (RPE) and different external (e.g., covered distance, speed, acceleration) and internal (e.g., HR) load parameters [14, 15]. These data suggest both external and internal load measurements should be considered for training management [15]. However, most of these studies have primarily examined the effects of soccer training programs on external and/or internal load, as well as the possible relationship between internal and external loads, among adult professional soccer players. A paucity of data is available in youth athletes, who require special consideration given that they not only train for soccer, but go to school during the week. Therefore, we investigated whether a six-week preparation phase increases physical performance and generates physiological adaptations in junior soccer players. Furthermore, we examined the relationship between external and internal stress parameters.

## MATERIALS AND METHODS

**FIG. 1:**
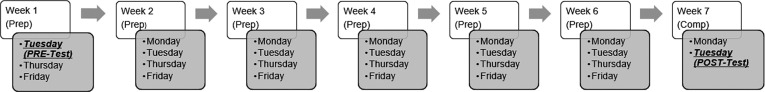
Schedule of the data collection during six-week football training. Prep: Preparation phase; Comp: competition phase.

An observational study was carried out to evaluate the development of subjective and objective load parameters in youth soccer players (i.e., 16 years old) following a six-week preparation phase. The Polar Team Pro GPS system from Polar Electro GmbH (Büttelborn, Germany) and a Wellness-Questionnaire from the Science on Field GmbH in Leipzig were used to measure load parameters. Data were collected in the first week of the preparation phase and again six weeks later, during the first week of the competitive phase during the 2020/2021 season. Both test sessions were part of the training program and were programmed on the same day of the week (Tuesday) and the same time of the day (afternoon) to minimize the effect of diurnal biological variations [16–18]. Tuesday was chosen as the control day in each case, as the soccer conditioning training took place on this day in both weeks (see Figure 1).

### Participants

A total of 19 soccer players from a U17 junior training centre were recruited to participate in the present study. Due to the Covid-19 pandemic and the associated restrictions as well as illnesses and/or injuries, 10 participants poorly adhered to the training program and were excluded. At the end of the study period, data from nine male players (age: 16 years old) were analysed and included in the final results.

After providing a detailed explanation about the study aims, design, and benefits, written informed consent was obtained from the parents. The protocol was approved by the local review board and the study was conducted according to the declaration of Helsinki.

### The six-week preparatory training program

Participants reported to the sport club four times per week (i.e., Monday, Tuesday, Thursday and Friday) to complete their assigned training program with a game scheduled each weekend. In the first two weeks, the focus was on ball possession with high volume and low intensity to improve football fitness [11]. On the third week, the focus was on losing the ball. Then, on the fourth week more defending exercises were programmed with a simultaneous increase in intensity and decrease in volume. The last two weeks training program focused on winning the ball with continuing increase in intensity and decrease in volume [11]. This set-up aims to progressively involve the players in high intensity exercises in order to improve their ability to perform more football-specific actions per minute with reduced risk of injury [11]. All training sessions consisted of football-based games, which, by changing the number of players, size of the pitch and work-rest ratio, targeted different training goals [11].

### Measurements

*The wireless Polar Team Pro* was used for this investigation. Sprint score, acceleration score, total distance, distance/min, maximum speed, average speed, and duration were examined as physical performance parameters; maximal HR, average HR, average R-R interval, maximal R-R interval, heart rate variability (HRV), and time in heart rate zones 4+5 were examined as physiological parameters. Training load score was also examined using this system.

The sensors of the system include an integrated GPS (measures at 10 Hz), a 10 Hz MEMS motion sensor, a 200 Hz accelerometer, and a sensor to measure the HR [21]. These sensors are attached to a chest strap, which is placed on the xiphoid process of the sternum with the chest strap fitted around the participant’s chest [22]. The Polar chest strap has been previously validated against the electrocardiogram gold standard and collects and processes HR measurements by detecting the electrical signals of the heart [23].

Each player received a sensor from Polar before the training session, which they wore for the entirety of the training session [24]. Each player received the same sensor on all training days to reduce measurement error [25, 26]. Table 1 shows detailed descriptions of the physical performance and HR parameters selected from the Polar Team Pro system.

**TABLE 1:** Selected physical performance and heart rate data from the Polar team

	Physical Performance parameters
Sprint score	A sprint is counted as a sprint when the player reaches an acceleration of 2.8 m/s^2^ regardless of the duration or distance of the sprint
Acceleration score	Summary of the number of negative and positive accelerations from 2 m/s^2^ in a training session
Total distance (m)	Total distance during the training session. (in meters).
Distance / min (m/min)	Average distance per minute during the training session. (in meters).
Maximum speed (km/h)	Maximum speed during the training session. (kilometre/hour)
Average speed (km/h)	Average speed during the training session. (kilometre/hour)
Duration (min)	Total training duration in min, starts with entering and ends with leaving the court

	**Physiological parameters**

Maximal heart rate (HRmax)	Maximal heart rate during the training session calculated as “beat/minute” and as % of the maximal heart rate (% HR max).

Average heart rate (HRavg)	Average heart rate during the training session calculated as “beat/minute” and as % of the maximal heart rate (% HR max).

Average RR interval (Avg RR)	Average beat-to-beat interval during the training session (millisecond). An increase over time means that fitness is improving.

Maximum RR interval (Max RR)	Maximum time between successive heartbeats (beat-to-beat interval) recorded during the training session (milliseconds).

HRV (RMSSD)	The root mean square of successive differences between normal heartbeats (RMSSD) is obtained by first calculating each successive time difference between heartbeats in milliseconds. Each of the values is then squared and the result is averaged before the square root of the total is obtained. The RMSSD reflects the beat-to-beat variance in heart rate and reflects short-term heart rate variability HRV [19]. High HRV is related to improved health and indicates that the heart is functioning well, and that the autonomic nervous system is adapting to the demands placed on it [20].

Time in HR zone 1	Heart rate (HR) zones are a way to monitor how the training intensity is. There are five heart rate zones based on the intensity of training with regard to the maximum heart rate. The % of the maximum heart rate in each zone are as following: Zone 1: 50–60%, Zone 2: 60–70%, Zone 3: 70–80%, Zone 4: 80–90%, Zone 5: 90–100% [21]. In the present paper, the time in heart rate zone 4 and 5 was added, because these two zones run in anaerobic energy supply
Time in HR zone 2	
Time in HR zone 3	
Time in HR zone 4	
Time in HR zone 5	

	**Training Load**

Training load score	Training Load includes textual feedback on the strenuousness of a single training session. It is based on intensity and duration of a training session with the intensity of a session is measured using HR and the calculation is further affected by personal information, such as age, sex, weight, VO_2max_, and training history. As participant’s fitness improves, same training session creates less training load (Polar).

*The Wellness-Questionnaire* was collected with the help of a webapp especially developed for this club. This application was developed by Science on Field GmbH in Leipzig and is based on a simple questionnaire system in which the players answer ten wellness questions on a 0–10 scale each morning.

The questionnaire contained 10 questions. The following questions were relevant for this study: “How is your muscle status today?” and “How was the training intensity the day before? The question “How was the training intensity on the previous day” corresponds to the question about the RPE from the previous day’s training. The authors of this paper did not have access to the data of all other eight questions of the application, as these are subject to the data protection of the junior training centre.

After each training session, the current muscle condition and training intensity of the previous day was recorded using the aforementioned web app. Players were required to complete the survey by 12:00h.

### Statistical Analysis

Data are presented as mean ± standard deviation (SD) and were analyzed using the Jamovi software (1.8.1, https://www.jamovi.org/). Descriptive statistics (mean ± SD) was calculated for muscle status, training intensity of the day before, number of sprints, accelerations, total distance, distance/min, maximum speed, average speed, duration, maximal HR, average HR, time spent in 80–100% the maximum HR, and the training load.

For the six-week training period, the Shapiro-Wilk test was used to assess data normality. Paired t-tests were performed to compare normally distributed data collected during the first vs. the second test session and Wilcoxon test was performed for non-normal distribution. Also, mean difference with 95% Confidence Interval (95%CI) and the effect size using Cohen’s d with 95%CI was calculated and interpreted as follows: < 0.2 as trivial, 0.20–0.59 as small, 0.60–1.19 as moderate, 1.20–1.99 as large and ≥ 2.0 as very large [27].

For correlations, the normality of the pooled values of all training units over the six weeks was checked with the Shapiro-Wilk test. Pearson correlation was performed for bi-normally distributed data and Spearman correlation was performed for non-normal distribution. The Rho-value was calculated as the Pearson’s and Spearman’s correlation effect size and interpreted as < 0.1 – trivial; 0.10 to 0.29 – small; 0.30 to 0.49 – moderate; 0.50 to 0.69 – large; 0.70 to 0.89 – very large; ≥ 0.90 – nearly perfect [27]. Significance for all analyses was accepted at the level of p < 0.05.

In order to calculate the gain or decrease over the six-week preparation period, difference scores (Δ) from PRE- (T1) to POST- (T2) test session (Δ = T2–T1) were calculated for all parameters. Additionally, based on tests and re-tests [28], the standard error of measurement (SEM) was established for HR- and HRV-related parameters, as well as for physical performance and strenuousness indicators, as previously described [29, 30]. Two times the SEM is considered the threshold for a true physiological adaptation beyond what would be expected from technical and/or biological variability [30, 31]. Therefore, responsiveness to the six weeks’ physical preparation training was defined as changes exceeding two times the SEM in favor of beneficial changes. The responsiveness threshold in each selected parameter, as well as the prevalence of responders and non-responders to training and detraining, have been reported in the results section.

## RESULTS

Table 2 shows the mean and standard deviation of all tested parameters measured during the PRE- and POST-test sessions, as well as the six week’s averaged values.

**TABLE 2:** Mean and standard deviation (SD) of the examined parameters

Development over six weeks				
	Parameter	PRE-test session	POST-test session	six weeks’ averaged values
Physical performance	Sprint score	19.44 ± 5.93	26.44 ± 4.09	12.55 ± 8.82
	Acceleration score	392.44 ± 30.6	458.11 ± 47.51	297.11 ± 92.69
	Total distance (m)	6847.22 ± 286.26	8099.44 ± 911.71	4763.21 ± 2139.98
	Distance/min (m/min)	63.67 ± 2.58	67.67 ± 7.48	51.49 ± 16.94
	Maximum speed (km/h)	29.29 ± 1.27	30.71 ± 1.42	26.24 ± 4.13
	Average speed (km/h)	4.32 ± 0.2	4.36 ± 0.45	4.05 ± 1.36
	Duration (min)	107.42 ± 0	120.0 ± 0	91.72 ± 19.59

Physiological responses	Maximal heart rate (beats/min)	192.33 ± 6.18	199.67 ± 17.03	189.11 ± 16.97
	Maximal heart rate (%)	96.33 ± 2.94	100.56 ± 7.96	95.25 ± 8.29
	Average heart rate (beats/min)	151.33 ± 5.77	144.56 ± 8.93	145.72 ± 17.60
	Average heart rate (%)	75.89 ± 2.85	73.0 ± 4.14	73.52 ± 8.79
	Average RR interval (msec)	404.89 ± 16.86	431.22 ± 35.23	425.22 ± 52.37
	Maximum RR interval (msec)	2019.56 ± 659.05	2620.67 ± 846.01	2369.80 ± 781.07
	HRV (msec)	9.89 ± 4.51	12.0 ± 5.83	11.11 ± 6.68
	Time in heart rate zone 4 + 5 (min)	39.18 ± 6.45	37.57 ± 9.10	25.17 ± 20.25

Training load score (AU)		192.44 ± 25.4	202.11 ± 39.73	131.40 ± 64.38
Muscle status		6.67 ± 1.89	7.0 ± 1.49	7.48 ± 1.60
RPE of the previous day		3.89 ± 2.02	3.89 ± 2.18	5.38 ± 2.70

### The effect of six-week preparation period on physical performances

**FIG. 2:**
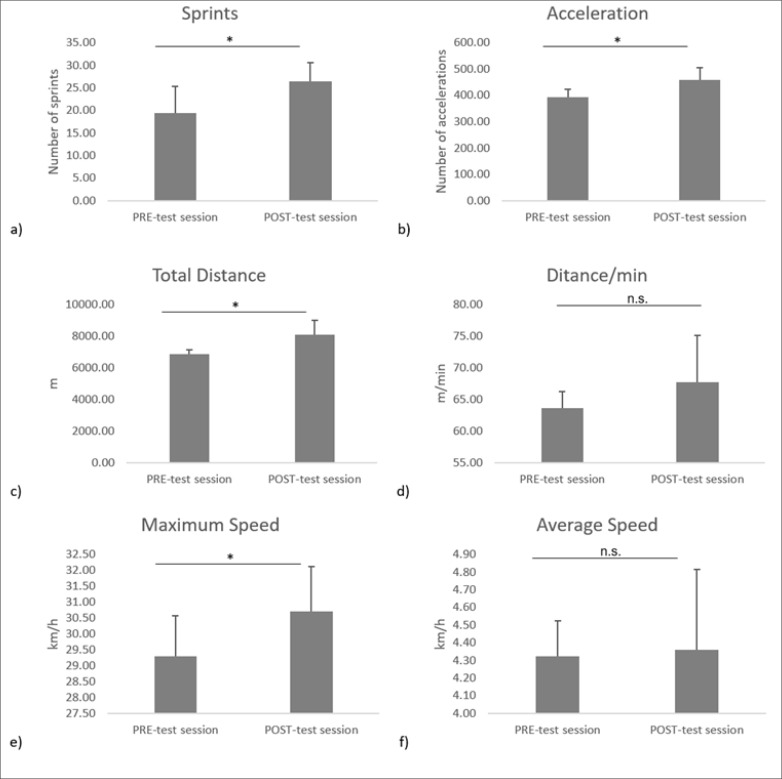
Mean ± SD values of a) number of sprints, b) accelerations, c) total distance, d) distance/min, e) maximum speed, and f) average speed at PRE- and POST-test session, *: significant difference between PRE- and POST-test sessions with p ≤ 0.05; n.s: means no significant difference between PRE- and POST-test sessions.

The values of physical performance parameters at PRE- and POST-test sessions are presented in Figure 2. Statistical analysis revealed significantly higher values at POST-test compared to PRE-test for number of sprints [p = 0.015, ES = 1.02 (moderate), mean difference = 7 (1.74;12.3)], acceleration score [p = 0.014, ES = 1, mean difference = 74.1 (53;96.5)], total covered distance [p = 0.02, ES = 0.87, mean difference = 1573 (191;1878)], and maximum speed [p = 0.02, ES = 0.87, mean difference = 1.4 (0.37;2.85)]. There were slight, non-significant increases in distance/ min [ES = 0.56, mean difference = 6.5 (-5;9)] and average speed [ES = 0.6, mean difference = 0.17 (-0.59;0.3)] from PRE- to POST-test session (p > 0.05).

### The effect of the six-week preparation period on physiological responses

**FIG. 3:**
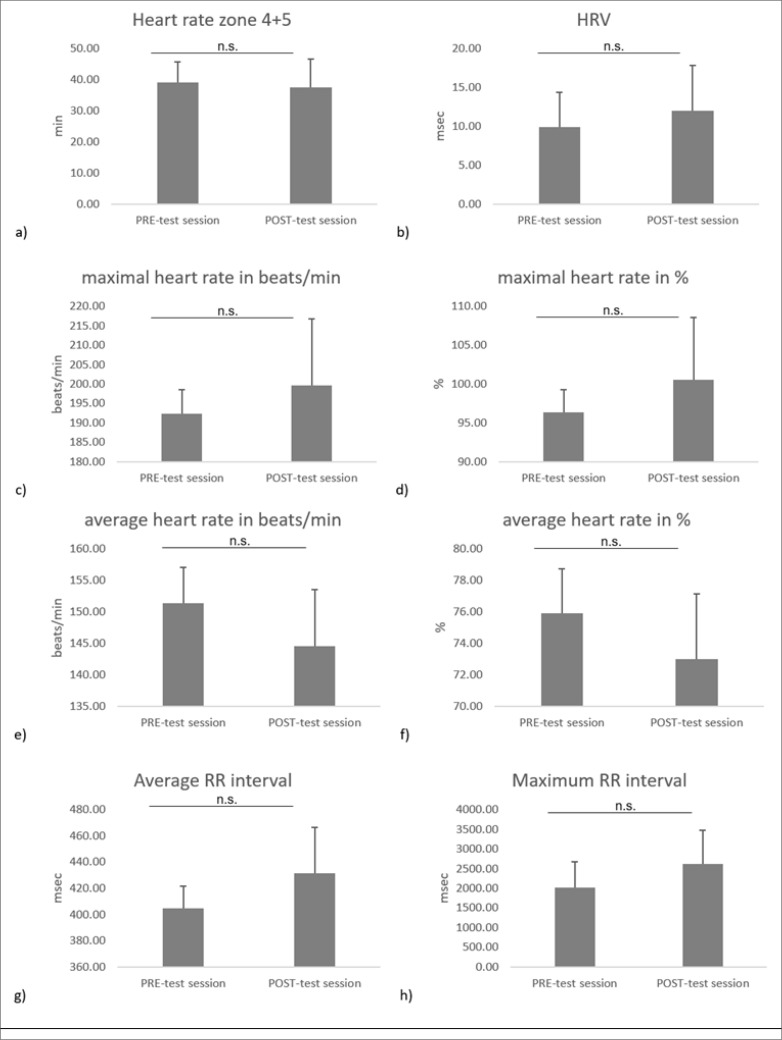
Mean ± SD values of a) heart rate zone 4+5, b) HRV c) maximal heart rate in beats/min, d) maximal heart rate in %, e) average heart rate in beats/min f) average heart rate in %, g) average RR interval, and h) maximal RR interval at PRE- and POST-test session. n.s: means no significant difference between PRE- and POST-test sessions.

Following the six-week preparation period, slight non-significant increases in HRV [p = 0.31, ES = 0.4, mean difference = 3 (-3;7)], maximal HR [p = 0.22, ES = 0.44, mean difference = 7.33 (-5.41;20.1) and p = 0.14, ES = 0.54, mean difference = 4.22 (-1.79;10.2) when expressed as beats/min and as %, respectively] and average [p = 0.09, ES = 0.64, mean difference = 26.3 (-5.37;58)] and maximal [p = 0.16, ES = 0.56, mean difference = 562 (-225;1399)] R-R intervals were measured during the POST- compared to the PRE-test session. In contrast, a slight non-significant decrease was registered following the six-week preparation period for time spent in HR zone 4+5 [p = 0.7, ES = 0.13, mean difference = -1.62 (-11;7.74)] and the average HR [p = 0.12, ES = 0.57, mean difference = -6.78 (-15.8;2.26) when expressed as beats/min and p = 0.17, ES = 0.5, mean difference = -2.89 (-7.33;1.55) when expressed as %]. The values of the physiological responses are shown in Figure 3.

### The effect of the six-week preparation period on training load and wellness parameters

**FIG. 4:**
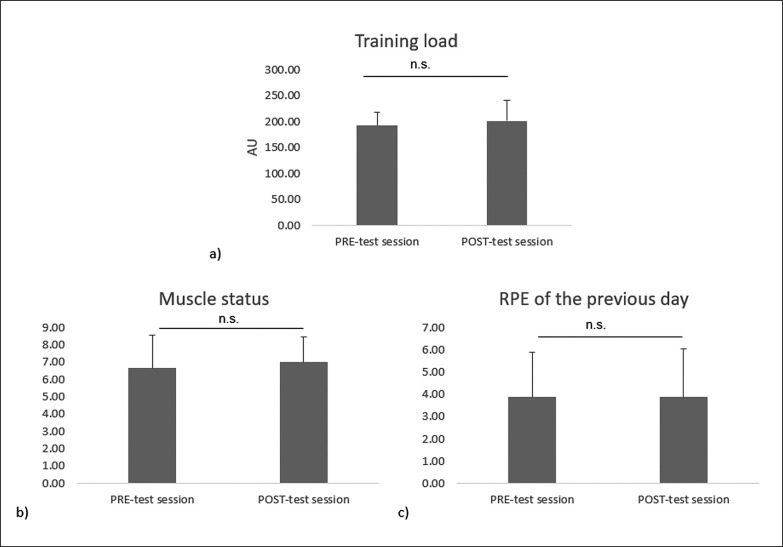
Mean ± SD values of a) training load, b) muscle status, and c) RPE of the previous day at PRE- and POST-test session. n.s: means no significant difference between PRE- and POST-test sessions.

Statistical analysis showed slight no significant increase in training load [p = 0.52, ES = 0.23, mean difference = 9.67 (-23;42.4)] and muscle status [p = 0.345, ES = 0.6; mean difference = 1 (0;1.5)] from PRE- to POST-test. Additionally, mean values of the RPE of the previous day were unchanged from PRE- to POST-test [p = 1.00, ES = 0, mean difference = -0.000042 (-0.5;1)]. The results are presented in Figure 4.

### Responsiveness of the U17 soccer players to the six-week preparation period

**FIG. 5:**
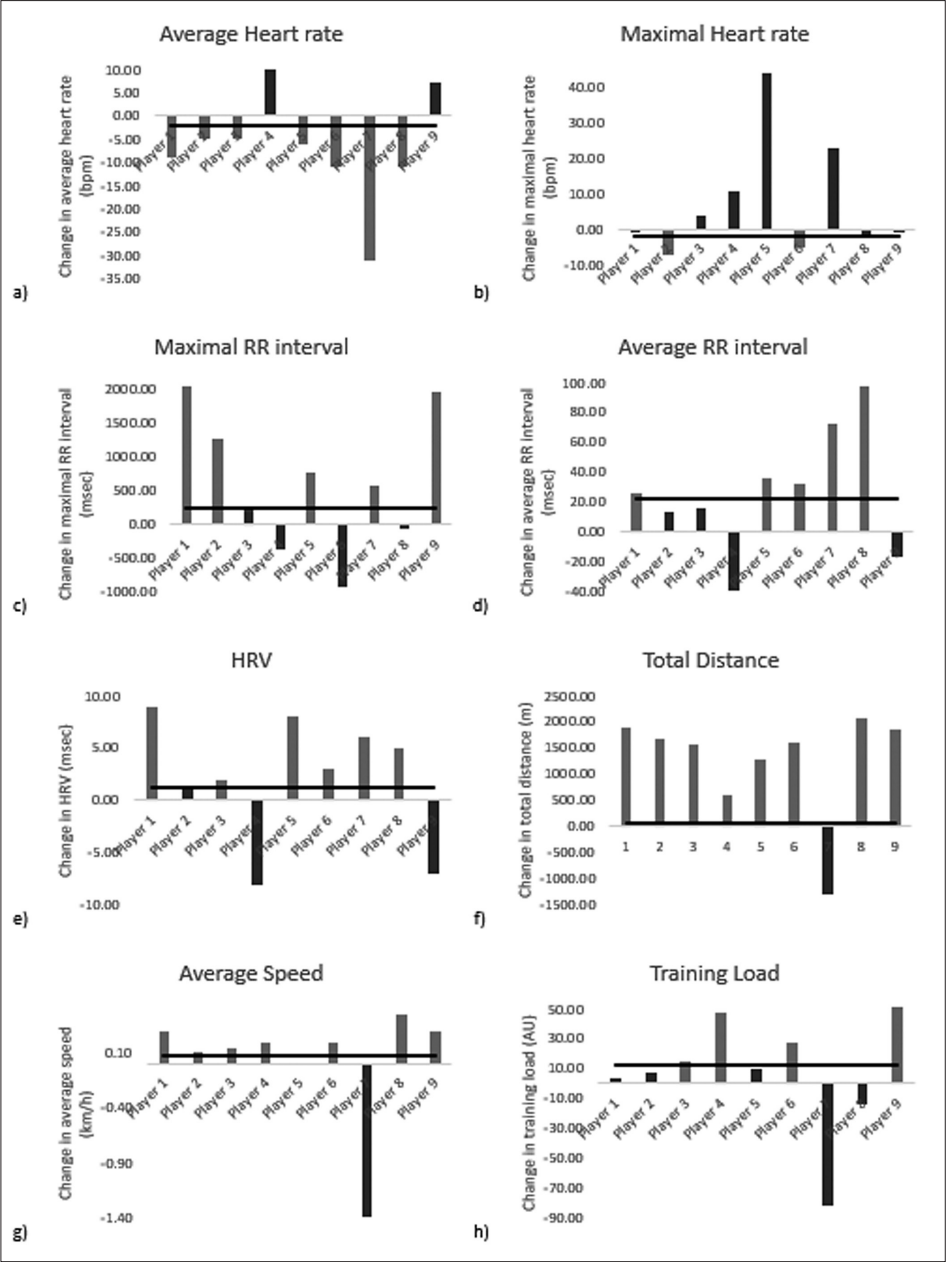
Individual responsiveness scores to six-week training period.

The prevalence of responders and non-responders to the six-week preparation training period are presented in Table 3. At least 2/3 of the players have been classified as responders to the beneficial training adaptation for total distance, average speed, average HR and HRV, while only 2 of the 9 players responded to the maximal HR training adaptation. For R-R and load score adaptations, approximately half of the players were not responders. Individual responsiveness scores can be found in supplementary materials (Figure 5).

**TABLE 3:** Prevalence of responders and non-responders to training adaptations.

Responsiveness to Training Adaptations								
Participants Number	HR (bpm)		HRV Related Parameters (msec)			Physical Performance and Strenuousness Indicators		
	HR Avg	HR Max	Max RR	Avg RR	HRV (RMSSD)	Total Distance(m)	Avg Speed (km/h)	Load Score
RT	-2.26	-2.01	241.85	22.01	1.3	71.16	0.07	11.9
Responders	6	2	5	5	6	8	7	4
Non-Responders	3	7	4	4	3	1	2	5
Non-responders %	33.33	77.78	44.44	44.44	33.33	11.11	22.22	55.56

### Relationship between external and internal loads in U17 soccer players

The correlation between the external and internal loads over the six weeks preparation training period (pooled values) are presented in Table 4.

**TABLE 4:** Correlation between external and internal loads when considering pooled values

Variables		Training Load, muscle status and RPE				Physiological Response						
		Training load	Muscle status	RPE of the previous day	Maximal HR beats/min	Maximal HR %	Average HR beats/min	Average HR %	Average RR interval	Maximum RR interval	HRV	Time in heart rate zone 4+5
**Physical performance**	**Sprint score**	**p < 0.001; r = 0.686**	p = 0.007; r = -0.176	P < 0.001; r = -0.379	**p < 0.001, r = 0.537**	**p < 0.001; r = 0.517**	**p < 0.001; r = 0.518**	**p < 0.001; r = 0.5**	p < 0.001; r = -0.464	NS	p = 0.007; r = -0.175	**p < 0.001; r = 0.677**

	**Acceleration score**	p < 0.001; r = 0.435	NS	NS	NS	NS	NS	NS	NS	p < 0.001, r = 0.266	NS	p < 0.001; r = 0.281

	**Total distance**	**p < 0.001; r = 0.834**	p = 0.012; r = -0.165	P < 0.001; r = -0.245	**p < 0.001, r = 0.509**	**p < 0.001; r = 0.517**	**p < 0.001; r = 0.536**	**p < 0.001; r = 0.541**	p < 0.001; r = -0.491	p < 0.001; r = 0.261	p < 0.001; r = -0.227	**p < 0.001; r = 0.818**

	**Distance/min**	**p < 0.001; r = 0.726**	NS	p = 0.002; r = -0.198	p < 0.001, r = 0.341	p < 0.001; r = 0.348	p < 0.001; r = 0.354	p < 0.001; r = 0.351	p < 0.001; r = -0.307	p < 0.001; r = 0.253	p < 0.001; r = -0.247	**p < 0.001; r = 0.645**

	**Maximum speed**	**p < 0.001; r = 0.603**	NS	p = 0.009; r = -0.17	p < 0.001, r = 0.443	p < 0.001; r = 0.429	**p < 0.001; r = 0.569**	**p < 0.001; r = 0.56**	**p < 0.001; r = -0.576**	NS	p = 0.001; r = -0.209	**p < 0.001; r = 0.66**

	**Average speed**	**p < 0.001; r = 0.535**	NS	P < 0.001; r = -0.217	p < 0.001, r = 0.471	p < 0.001; r = 0.491	**p < 0.001; r = 0.871**	**p < 0.001; r = 0.83**	**p < 0.001; r = -0.761**	NS	p < 0.001; r = -0.276	**p < 0.001; r = 0.743**

	**Duration**	p < 0.001; r = 0.458	NS	p = 0.016; r = -0.158	p < 0.001, r = 0.389	p < 0.001; r = 0.412	p < 0.001; r = 0.493	p < 0.001; r = 0.498	p < 0.001; r = -0.504	NS	NS	**p < 0.001; r = 0.579**

**Training load**		**1**	p = 0.01; r = -0.169	p < 0.001; r = -0246	**p < 0.001, r = 0.637**	**p < 0.001; r = 0.637**	**p < 0.001; r = 0.689**	**p < 0.001; r = 0.697**	**p < 0.001; r = -0.654**	p < 0.001; r = 0.247	p < 0.001; r = -0.234	**p < 0.001; r = 0.925**

**Muscle status**		p = 0.01; r = -0.169	**1**	**p < 0.001; r = 0.563**	p = 0.014 r = -0.16	p = 0.027; r = -0.145	p = 0.004; r = -0.189	p = 0.006; r = -0.18	p = 0.005; r = 0.185	NS	NS	p = 0.002; r = -0.202

**RPE of the previous day**		p < 0.001; r = -0.246	**p < 0.001; r = 0.563**	**1**	p = 0.004 r = -0.189	p = 0.008; r = -0.174	p < 0.001; r = -0.233	p < 0.001; r = -0.225	p < 0.001; r = 0.241	NS	p < 0.001; r = 0.221	p < 0.001; r = -0.255

The majority of the physiological responses showed a significant correlation (p < 0.001) with all physical performance indicators, except acceleration. Importantly, Time in HR zone 4+5 showed moderate to large correlations (p < 0.001; 0.58 < r < 0.82) with all physical performance indicators, except acceleration (trivial correlation); average HR (i.e., beat/min or %) showed moderate to large correlations (p < 0.001; 0.50 < r < 0.87) with four physical performance indicators; maximal HR (beats/min) as well as average R-R interval showed moderate to large correlations (p < 0.001; 0.51 < r < 0.76) with two physical performance indicators. However, maximum R-R interval and HRV showed only trivial (p < 0.001; 0.18 < r < 0.28) or non-significant correlation (r > 0.5) with the physical performance indicators. It should be also noted that training load was significantly correlated with all physical performance indicators and physiological responses with p < 0.001 and r > 0.5 in the majority of these correlations. However, the correlation between RPE of the previous day and the majority of the physical performance and physiological responses were only trivial to small (p < 0.001; 0.5 < r < 0.82). Muscle status showed also trivial correlations (p < 0.001; 0.5 < r < 0.82) or no-significant correlation (r > 0.5) with the physical performances and physiological responses over the six weeks training period.

## DISCUSSION

The aim of this study was to examine the effects of a six-week preparatory training on physical performance and physiological adaptations in junior soccer players. Furthermore, it was investigated whether relationships existed between external and internal loads and between objective and subjective internal loads in professional youth soccer players. The main findings included significant increases in the majority of physical performance indicators, and a non-significant trend towards improvements in training load, muscle status and all physiological responses parameters. Furthermore, significant correlations were found between the majority of external load parameters (i.e., performance indicators) and objective (i.e., physiological responses) and subjective (i.e., RPE) internal load parameters and training load. The highest number of moderate-large correlations were registered between performance indicators and the time in HR zone 4+5, average HR, maximal HR, average R-R interval and training load.

Improvements in the majority of physical performance parameters (i.e., sprints, acceleration, total distance and max speed) following the six-week preparation training program are in line with previous findings of Lee and Joo [32] and Asadi et al. [33], who reported improvements in fitness levels and/or sprint ability after eight and six weeks, respectively during preseason training in youth soccer players. Ehrmann et al. [34] found an increase in distance/min (an indicator of workload intensity [35]) was associated with an increased risk of injury. Therefore, it can be assumed that the present preparation training program did not generate an increased risk of injury to the players as the distance/min did not increase significantly. Additionally, the higher values of maximum speed, sprints and accelerations during the POST-test compared to the PRE-test session, indicates the youth soccer players were able to perform more soccer-specific maximal actions following the six-week training program. As the average speed remained significantly unchanged from PRE- to POST-test session, more slower actions must have taken place to compensate for these maximal actions indicating that youth soccer players may adopt more intermittent-based training following the six-week preparation training period.

Regarding the physiological responses, it is well known that a decrease in HR values (i.e., maximum and average HR), and an increase in HRV related parameters, (i.e., HRV and average and maximum R-R intervals), in response to similar training session following a training period, is considered as beneficial cardiac adaptation [30]. Present results indicated a tendency of (i) decrease in average HR and time in heart zone 4+5, and (ii) increase in maximal HR, average and maximal R-R interval and HRV, following the six weeks training program. These findings suggest six weeks of preparatory training may produce slight cardiac adaptations, except for the maximal HR, in the youth soccer players. These results are consistent with previous studies reporting that regular soccer training in the form of small games two to three times a week leads to significant cardiovascular adaptations, regardless of the participants training level, gender, and age [36]. School-based ten-week intervention in the form of small-sided, intermediate-level soccer training also led to structural and functional cardiac adaptations in pre-adolescent boys and girls [37].

The absence of significant improvements in the majority of the physiological responses following the present six weeks training program can be explained by the low responsiveness rate of the entire teams players. Our results indicate that, for all physiological responses, the responsiveness rate to the training adaptations did not exceed 66.7% (i.e., 66.7% for average HR and HRV adaptation, 55.6% for maximal and average R-R interval and only 22.2% for maximal HR); therefore, no significant changes were found for the whole team. However, looking at the responsiveness rate for total distance adaptation, we found a higher rate of nearly 89% and significant improvements for the entire team following the training program. These findings indicate youth soccer players did not adapt similarly, especially in terms of physiological responses, to the preparation training program. This suggests some of them may have experienced undertraining and/ or suboptimal conditioning due to imbalances in loads and recovery [38]. The content of future training programs should be more individualised and/or differentiated to increase the responsiveness rate and generate significant physiological improvement at the team level. This can be achieved by measuring individual changes in fitness and fatigue markers, which in turn allows quantification of individual response and adaptation to training. This will allow coaches to better understand the impact of programmed loads on players and identify personalized tolerance thresholds for each player [39]. Additionally, it seems imperative to monitor the training load that the athlete experiences during each training session to support training individualisation and avoid incorrectly dosed training load (undertraining or overtraining) [40]. Training monitoring allows the coach to compare planning and achievements and is specific to the athletes age, sex, and performance level [6]. Furthermore, training monitoring is understood as the visualisation and objectification of a training process with the help of suitable parameters and technical instruments [6]. In this context, and in order to determine the most suitable internal loads parameters for the monitoring process, the present study investigated also the relationship between performance indicators and a number of objectives and subjective internal training load parameters and the training load.

Overall, results showed significant correlations between the majority of the physical performance indicators and both objective (HR and HRV related parameters) and subjective (i.e., muscle status and RPE) internal training load parameters in youth soccer players. These findings are consistent with previous studies in professional soccer players showing that external load parameters such as total distance, low-, high- and very-high running distances and times [15], frequency of efforts at high speed [14], and various HR based training loads correlate significantly with RPE [4].

This suggests the highest number of moderate to large correlations were registered between performance indicators variables and the time in HR zone 4+5, average and maximal HR, average R-R interval, and training load. Taking into consideration that successful training monitoring should be very specific to the athlete’s performance level [6], these findings indicate HR zone 4+5, average and maximal HR, and average R-R interval as most suitable internal load parameters and training load itself for optimal monitoring process. Monitoring these variables may help the coach to implement correct load management, and reduce the risk of under- or over-training, as well as the risk of injury.

Previous studies also reported significant correlations between objective and subjective internal loads in professional soccer players [4, 15, 40]. Results from this study confirm this relationship in youth soccer players and showed that the majority of the HR and HRV related parameters were significantly correlated with muscle status and RPE. However, these correlations were only trivial to small with muscle status and RPE of the previous status. The present findings also further suggest a relationship between objective internal loads and training load in youth soccer players. These results identify training load as a potentially significant parameters to provide good information about the status of youth soccer players during the monitoring process.

In summary, both external and internal (objective and subjective) parameters and training load should likely be considered for training monitoring and individualisation of youth soccer players in order to optimise training planning, physiological adaptation and physical performance of the whole team.

### Strengths and limitations

The present study is the first to assess the relationships between (i) external and internal loads and (ii) objective and subjective internal loads in professional youth soccer during the pre-conditioning phase. However, there are several limitations warranting further discussion. First, the limited sample size and inclusion of only one age group (16 years old) may limit generalization of the present study. Second, the experimentation period was limited to the preparation phase of the season and only data of male youth soccer players were collected. Therefore, future studies should investigate larger samples size from both sexes and from different ages (15–18 years old) during different periods of the season (or the whole season) to draw more finite conclusions. Additionally, several methods have been proposed to analyse responsiveness rate to training program, but there is no consensus on which should be considered the gold standard. Thus, our findings concerning inter-individual variability should be interpreted considering this concept.

## CONCLUSIONS

In conclusion, these results suggest youth soccer players do not adapt similarly, especially in terms of physiological responses, to the six-week preparation training program. Therefore, the content of future training programs may require more individualization and/or differentiation to increase the responsiveness rate and generate significant physiological improvement in the whole soccer team. Additionally, when using the polar system, the time in HR zone 4+5, followed by training load, average HR and then maximal HR and average R-R interval may be considered an important monitoring variable in youth soccer players. Future research evaluating expanded sample sizes and sexes are required to confirm these findings.

## Competing interest statement

All authors declare no competing interest.

## Details of funding

The author received no specific funding for this work.
